# 

**Published:** 1895-07

**Authors:** 


Vol. XVI. JULY, 1895. No. 7.

PUBLISHED MONTHLY AT $3 A YEAR. SINGLE COPIES, 30 CENTS.

### THE JOURNAL OF COMPARATIVE MEDICINE AND VETERINARY ARCHIVES

EDITED AND PUBLISHED BY

RUSH SHIPPEN HUIDEKOPER, Veterinarian (Alfort),
W. A. CONKLIN, PH.D., D.V.S., A. W. CLEMENT, D.V.S.,
W. HORACE HOSKINS, D.V.S., H. D. GILL, V.S.

ALL COMMUNICATIONS AND PAYMENTS SHOULD BE ADDRESSED TO

**Dr. W. Horace Hoskins, 3452 Ludlow Street, Philadelphia, Pa.**

Copies may be had in London of Baillière, Tindall & Cox.

